# Tuberculosis in Sudan: systematic review and meta analysis

**DOI:** 10.1186/s12890-024-02865-6

**Published:** 2024-01-23

**Authors:** M M Badawi, M A SalahEldin, A B Idris, E B Idris, S G Mohamed

**Affiliations:** 1 Higher Academy for Strategic and Security Studies, Khartoum, Sudan; 2 Medical Microbiology Department, Faculty of Medical Laboratory Sciences, University of Khartoum, Khartoum, Sudan; 3 General Surgery Resident, Medical Specialization Board, Khartoum, Sudan; 4 Department of medical microbiology, Rashid Medical Complex, Riyadh, Saudi Arabia

**Keywords:** Africa, Developing countries, Respiratory infections, Risk factors

## Abstract

Every year, 10 million people fall ill with tuberculosis (TB). Despite being a preventable and curable disease, 1.5 million people die from TB each year -making it the world’s top infectious disease. TB is the leading cause of death of people with HIV and also a major contributor to antimicrobial resistance. Its presumed that TB was the cause of 1% of the total deaths among inpatients in Sudan in 2017. The current study is aimed to provide pooled prevalence of Mycobacterium tuberculosis among Sudanese as well as to determine any socio-cultural risk factors associated. A systematic review of the literature was conducted and regulated in accordance with the PRISMA Statement. After abstract and full text screening only twenty-six articles met our inclusion criteria and passed the quality assessment procedure. Pulmonary tuberculosis prevalence was assessed in sixteen included studies among participants from Khartoum, Gezira, Kassala, Blue Nile, River Nile, White Nile, Gadarif, Red sea, North Kordofan, Northern State, Sennar and West Darfur States, representing a total sample size of 11,253 participants of suspected individuals such as febrile outpatients, TB patients’ contacts and other groups such as HIV/AIDS patients, hemodialysis patients, School adolescents as well as pregnant women. The pooled prevalence was 30.72% [CI: 30.64, 30.81]. Moreover, Khartoum State recorded the highest pooled prevalence as 41.86% [CI: 14.69, 69.02] based on a total sample size of 2,737 participants. Furthermore, male gender and rural residence were found to be significantly associated with TB infection. Further research with larger sample sizes targeting prevalence and risk factors of TB among Sudanese population is needed to be conducted.

**Supplementary Information:**

The online version contains supplementary material available at 10.1186/s12890-024-02865-6.

## Introduction

Bearing in mind the political issues that have plagued Sudan with war and hostility for the last 25 years, health care has become an afterthought and basically lost in the midst of what the government might believe to be more pressing matters. The country faces escalating humanitarian catastrophe, with 7.8 million people facing critical problems related to mental and physical wellbeing, including 1.6 million internally displaced people and 1.1 million refugees. Resources are scarce, economic output is collapsed by two-thirds between 2017 and 2018 and the country’s health system is ill-equipped to respond to growing and neglected needs. Adding insult to injury, Sudan still has a long way to go to achieve the Sustainable Development Goals (SDGs). According to the WHO as well as the Sudan Health Observatory in the federal ministry of health, the major communicable diseases contributing to morbidity are Malaria, Tubercelosis, Schistosomiasis, Pneumonia and Diarrheal diseases [1, 2].

Every year, 10 million people fall ill with tuberculosis (TB). Despite being a preventable and curable disease, 1.5 million people die from TB each year -making it the world’s top infectious disease. TB is the leading cause of death of people with HIV and also a major contributor to antimicrobial resistance. Its presumed that TB was the cause of 1% of the total deaths among inpatients in Sudan in 2017 [3].The current study is aimed to provide pooled prevalence of Mycobacterium tuberculosis among Sudanese as well as to determine any socio-cultural risk factors associated.

## Materials and methods

### Search strategy

To identify relevant studies; a systematic review of the literature was conducted in the 1st of December 2022. The review was regulated in accordance with the PRISMA (Preferred Reporting Items for Systematic Reviews and Meta-Analyses) Statement [4] (Table S1). A comprehensive search was operated in PubMed, Embase, Google scholar, Scopus, Index Copernicus, DOAJ, EBSCO-CINAHL, Cochrane databases without language limits (studies written in languages other than English were later excluded). To obtain a current situation evidence; only studies published in or after 2010 were included. Furthermore, all studies where the data collection process took place before 2010 were also excluded, the only exception was if the collection process started in or before 2010 and ended in 2010 or afterwards. All studies were checked independently by each author, votes were given for each study and any discrepancy was resolved through discussion.

As medical literature in Sudan is generally scarce in international databases as well as socio-cultural factors may be differently reported, socio-cultural factors were not used in keyword formulation and their related results were later extracted from included studies, the keywords used in PubMed was as follow:

Tuberculosis[Mesh] OR Tuberculosis[tiab] AND Sudan*. As previously described [5].

Moreover, to optimize our search, hand searches of reference lists of included articles were also performed.

### Study selection and data extraction

Titles and abstracts were assessed for preliminary eligibility. A copy of the full text was obtained for all research articles that were available and approved in principle to be included. Abstraction of data was in accordance with the task separation method; method and result sections in each study were separately abstracted in different occasions to reduce bias. Moreover, data abstraction was conducted with no consideration of author’s qualifications or expertise as described in details previously [6]. Each research article was screened for all relevant information and recorded in the data extraction file (Microsoft Excel), data from each method section was extracted using a predefined set of variables; study characteristics, type of participants, study population size, geographical region, methodology used in prevalence or risk assessment and the period of the study conduction.

After inclusion, studies were further classified into studies determining prevalence, studies determining socio-cultural risk factors and studies determining both prevalence and socio-cultural risk factors. Furthermore, as risk factors-related keywords were not formulated in the search strategy, each study was fully screened to check the nature of the risk investigated by authors, studies determining risk factors in which socio-cultural risks have not been assessed, were later excluded.

Although age grouping is available alongside their corresponding included study in (Table 1), it was not possible to be included in the Meta analysis due to the complexity and diversity of categorization of ‘’age’’ variable among studies included.

### Assessment of quality

Each included article was evaluated based on a framework for making a summary assessment of the quality. The related published literature was crossed, then a framework was structured specifically to determine the level of representativeness of the studied population and to judge the strength of the estimates provided. Five questions were to be answered in each article, each answer represent either 1 score for yes, 0 score for No or 0 score for not available; a total score for risk of bias and quality was calculated by adding up the scores in all five domains, resulting in a score of between 0 and 5. The highest score indicates the highest quality, only studies with a score for quality greater or equal to 3 (higher quality) were included.

The five domains were: is the study objective clearly defined?, is the study sample completely determined?, is the study population clearly defined and specified?, is the methodology rigorous? And is the data analysis rigorous?

#### Secondary analysis

Among all included studies reporting either prevalence or risk factor estimates, articles were crossed whether Standard Error (SE) is reported. In studies where the *SE* is not reported; the following formula was used to calculate it: *SE* = √p (1-p)/ n, where *p* stands for Prevalence. Regarding risk factors, as each included study may have different objective influencing thereby their result demonstration (i.e. adjusted OR, unadjusted OR, frequencies), each individual category in a given socio-cultural variable investigated the Odd Ratio (OR) was calculated (whenever possible) to provide univariate analysis for the given category among investigated population.

Categorizations of variables was designed to increase the population size of a given estimate, i.e. when the majority of studies investigating Tuberculosis socio-cultural risks categorize education level as below secondary and secondary/above; primary, secondary and university categorization in the minority of studies was re-categorized as (Primary = below secondary, secondary and university = secondary/above).

### Quantitative analysis

Meta-analysis was performed—whenever possible using Review Manager Software (Version 5.3). The software automatically provided the Confidence Interval (CI) according to the calculated *SE*, if the *CI* is provided in a study; it was introduced accordingly. The heterogeneity of each meta-analysis was assessed as well, the random effect was favored over the fixed effect model in all meta-analysis established as variations between studies is predicted to be probable due to the diversity of the study populations. Sensitivity analysis was also approached to determine the effect of studies conducted in populations proposed to behave in indifference manners or proposed to have low risk on the overall pooled data. Moreover, subgroup analysis was also conducted -whenever suitable to determine prevalence or risk level in specific State or population. An outcome to take part in the meta-analysis has to be included in at least two studies.

Trim and Fill method was used to assess the risk of publication bias in each Meta analysis conducted [7].

## Results

### Studies included

**Fig. 1:**
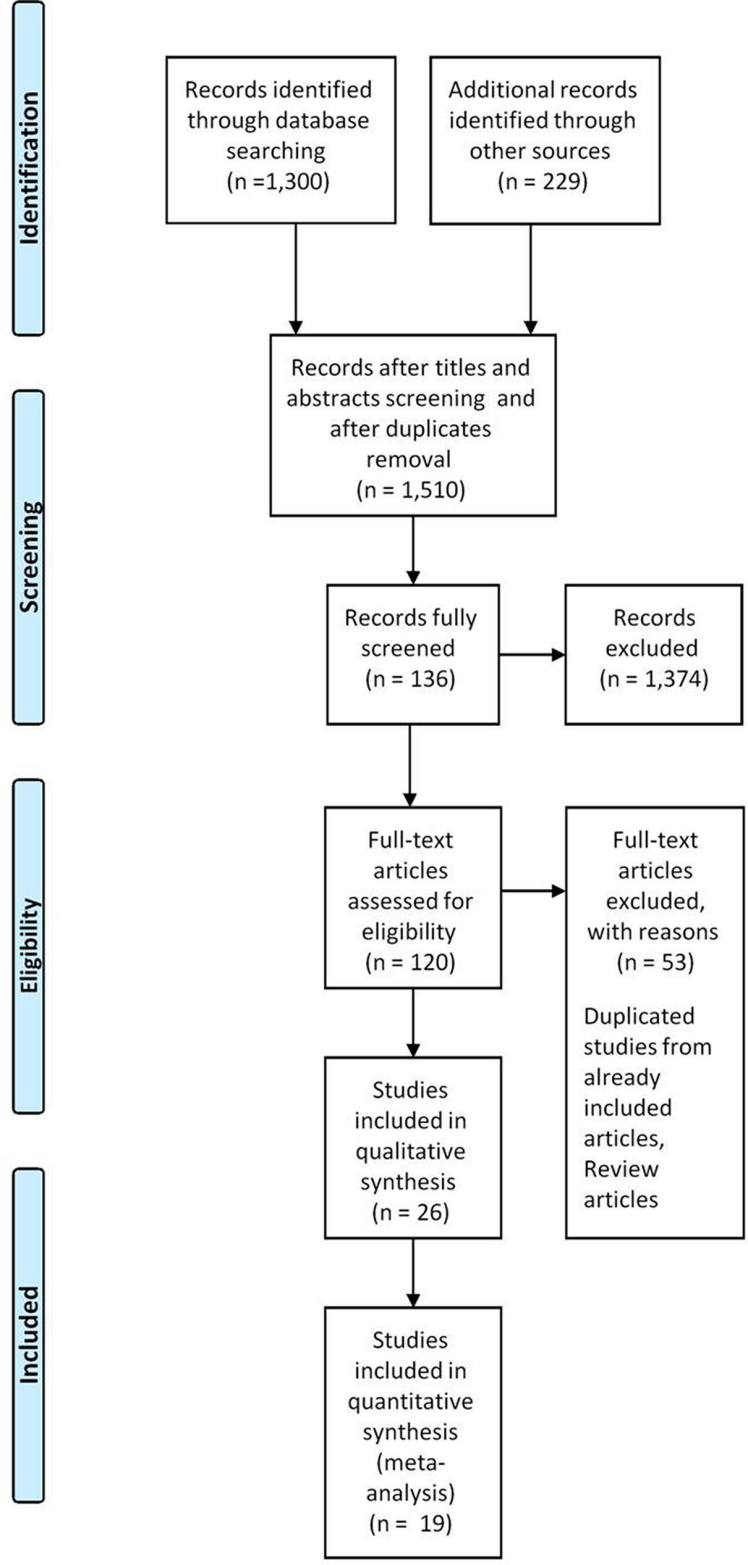
PRISMA flow diagram

A total of 1,510 articles were identified from the search strategy including hand searches of reference lists of included original research articles and reviews. From these, 1,371 articles were excluded. After abstract and full text screening only twenty-six articles met our inclusion criteria and passed the quality assessment procedure. The articles reported prevalence among specific population and/or risk factors. Figure 1 illustrates the PRISMA flow diagram. The included articles are depicted in (Table 1). Assessment of the quality of included studies is depicted in (Table S2).

### Study characteristics

The characteristics of the included studies are depicted in (Table 1), twenty-six studies were recruited to the study [8, 9, 10–19, 20, 21–26, 27–33], among which sixteen studies determined prevalence of pulmonary TB among Sudanese participants from different populations; participants were from Khartoum in seven included studies, from Kassala in three included studies, Blue Nile, River Nile, White Nile, Gadarif, Red sea, North Kordofan, Northern State, Sennar and West Darfur States were also participated, three studies only provided data related to prevalence of pulmonary and extra-pulmonary TB rather than general TB prevalence. Moreover, majority of studies were conducted among both genders, two studies were conducted among females, while one study did not determine the gender of their participants. Participant’s age varies among studies. All characteristics of included studies are depicted in (Table 1). Publication bias assessment indicated no major asymmetry.

**Table 1:** Characteristics of tuberculosis related studies

Study ID	Year of publication	Study design	State	study population/s	Assessment of	sample size	Gender	Participants’ Age
Abdallah, 2015 [8]	2015	Cross sectional	Kassala	Suspected Adults	Prevalence (Nuclic acid)	985	Both	Mean 34 years
Abdallah, 2012 (9)	2012	Cross sectional	Kassala	TB patients	Risk factors	670	Both	Not determined
Abdelhadi, 2015 [12]	2015	Case control	Kassala	TB patients	Risk factors	306	Both	15–85 years
Agab Eldour, 2014 [19]	2014	Retrospective	Kordofan	Suspected TB patients	Prevalence (Histopathological methods)	103	Both	13–65 years
Mustafa, 2021 [33]	2021	Cross sectional	Kassala	TB patients	Risk factors	251	Both	4–80 years
Ali, A. A., 2012 [21]	2012	Cross sectional	Kassala	Suspected TB patients	Prevalence & Risk factors	2,778	Female	Not determined
Ali, A. 2016 [22]	2016	Case control	Khartoum	TB patients	Risk factors	315	Both	Mean 33 years
Aman, 2017 [23]	2017	Case control	Gezira	TB patients’ contacts	Prevalence (Bacteriological and radiological methods)	657	Both	Mean 33 years
Awadalla, 2010 [24]	2010	Case control	Khartoum	HIV/AIDS patients	Prevalence	291	Both	Mean 36 years
Banaga, 2016 [25]	2016	Cross sectional	khartoum	hemodialysis patients	Prevalence & Risk factors	1,328	Both	Mean 36 years
Bottieau, 2022 [26]	2022	Cross sectional	Gadarif	Febrile patients	Prevalence (Bacteriological and Nuclic acid methods)	667	Both	Media 35 years
Elamin, 2017 [20]	2017	Cross sectional	Blue Nile	Suspected TB patients	Prevalence & Risk factors	208	Both	5–54 years
Elhassan, 2011 [28]	2011	Cross sectional	Khartoum	Suspected TB patients	Prevalence (Nuclic acid)	90	Not determined	Not determined
Elhassan, 2016 [27]	2016	Cross sectional	Khartoum	Suspected TB patients	Prevalence (Bacteriological methods)	197	Both	< 15 years
Elmadhooun, 2017 [29]	2017	Secondary analysis	River Nile	TB patients	Prevalence (Bacteriological methods)	187	Both	< 15 years
El-Muttalut & Elnimeiri, 2017 [30]	2017	Cross sectional	Kassala	TB patients	Risk factors	366	Both	10 - > 60 years
Ismail, 2016 [31]	2016	Retrospective	Gezira	TB patients	Risk factors	839	Both	< 18 - > 45 years
Khalid, 2020 [32]	2020	Cross sectional	Kassala	School adolescents	Prevalence (Bacteriological methods)	2,568	Both	5–15 years
Osman, 2014 [10]	2014	Cross sectional	Khartoum	Suspected TB children	Prevalence (Bacteriological methods & Nuclic acid)	179	Both	Median 8 years
Osman, 2017 [11]	2017	Cross sectional	Eastern Sudan^*^	TB patients’ contacts	Prevalence (Bacteriological methods & ELISA	768	Both	Mean 33 years
Saeed, 2021 [13]	2021	Cross sectional	River Nile	TB patients	Risk factors	212	Both	15 - > 45 years
Shakak, 2013 [14]	2013	Cross sectional	Khartoum	TB patients’ contacts & General population	Prevalence (Bacteriological methods & ELISA	284	Both	Not determined
Shigidi, M., 2012 [15]	2012	Retrospective	Khartoum	hemodialysis patients	Prevalence (Bacteriological methods & Nuclic acid) & Risk factors	350	Both	Mean 37 years
Shuaib, 2018 [16]	2018	Cross sectional	Kassala, Red Sea & Gadarif	Suspected TB patients	Prevalence (Bacteriological methods & Nuclic acid)	385	Both	Median 35 years
Sirelkhatim, 2016 [17]	2016	Cross sectional	Khartoum, Red Sea & North Kordofan	Suspected TB patients	Prevalence (Bacteriological methods) & Risk factors	243	Both	< 15–70 years
Yassin, 2019 [18]	2019	Cross sectional	Kassala	Pregnant women	Prevalence (Bacteriological methods) & Risk factors	149	Female	Mean 30 years

*Further details are not available

### Tuberculosis prevalence

Prevalence estimates were synthesized to represent the overall burden of the disease as well as to estimate subgroup burden related to disease type, study population and geographic location–whenever possible as illustrated below, summary of prevalence estimates synthesized from Tuberculosis related included studies is available in (Table 2).

#### Tuberculosis prevalence among different populations

**Fig. 2:**
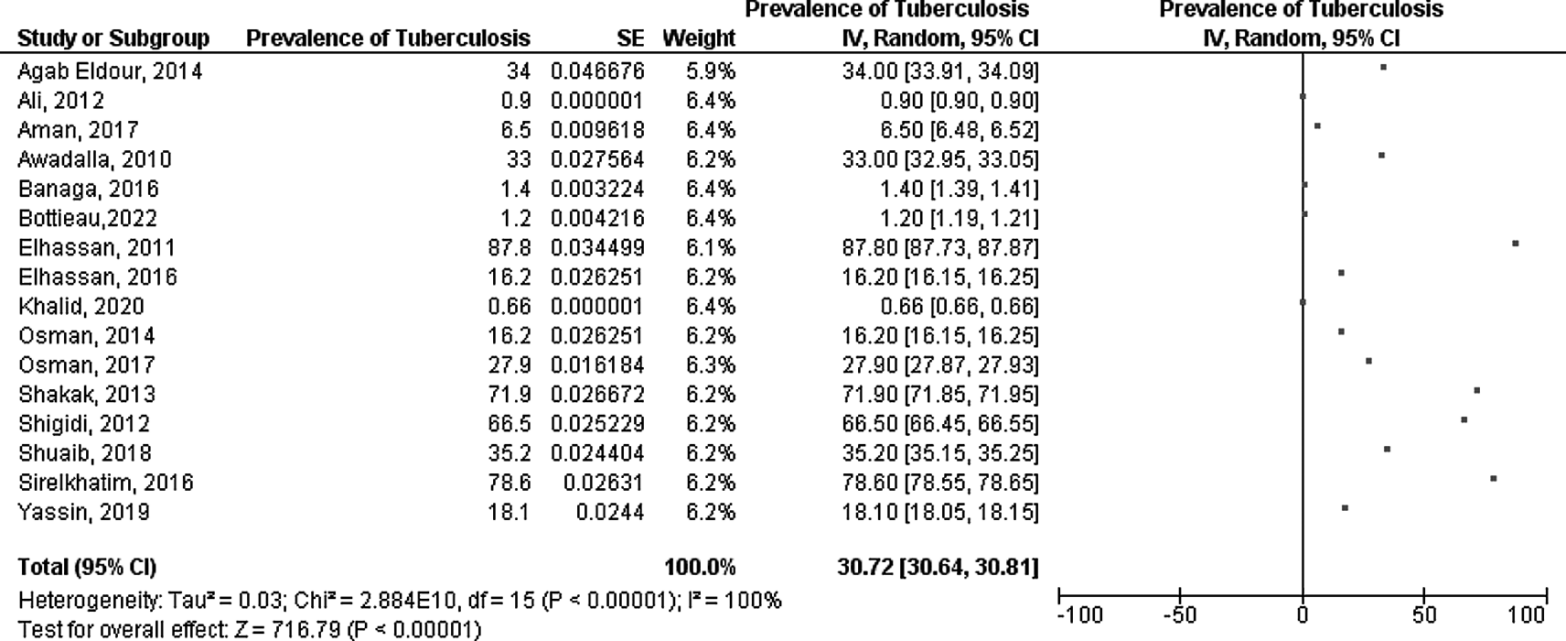
Meta analysis of prevalence of TB among participants

Nineteen included studies determined prevalence of TB among Sudanese participants from different populations, among which three studies only provided data related to prevalence of pulmonary and extra-pulmonary TB rather than general TB prevalence. Pulmonary tuberculosis prevalence was assessed in sixteen included studies; studies were conducted in Khartoum, Gezira, Kassala, Blue Nile, River Nile, White Nile, Gadarif, Red sea, North Kordofan, Northern State, Sennar and West Darfur States, representing a total sample size of 11,253 participants of suspected individuals such as febrile outpatients, TB patients’ contacts and other groups such as HIV/AIDS patients, hemodialysis patients, School adolescents as well as pregnant women. All characteristics are depicted in (Table 1). The pooled prevalence was 30.72% [CI: 30.64, 30.81]. After conducting sensitivity analysis the pooled prevalence was 28.74% [CI: 25.76, 31.72]. However, heterogeneity was high (I^2^ = 100%) (Fig. 2).

### Extra-pulmonary TB prevalence

**Fig. 3:**
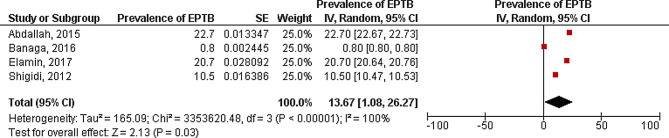
Meta analysis of prevalence of Extra-Pulmonary TB among participants

Extra-pulmonary TB prevalence was assessed among participants in four included studies. Studies were conducted among suspected adults in Kassala, Gadarif as well as Blue Nile States and among hemodialysis patients in Khartoum State [26], representing a total sample size of 2,871 participants from both genders with majority of participants being in their thirties. The pooled prevalence was 13.97% [CI: 1.08, 26.27]. Heterogeneity was high (I^2^ = 100%) (Fig. 3).

### TB prevalence among specific populations

#### TB prevalence among patients’ contacts

**Fig. 4:**
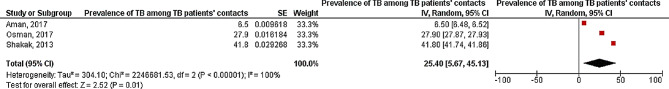
Meta analysis of prevalence of TB among TB patients’ contacts

TB prevalence among patients’ contacts was assessed among participants in three included studies. Studies were conducted in Eastern Sudan without State specification in one included study as well as Khartoum and Gezira States, representing a total sample size of 1,709 participants from both genders with participants’ age mean as 33 years in two studies while it was not determined among participants of the third study. The pooled prevalence was 25.40% [CI: 5.67, 45.13]. Heterogeneity was high (I^2^ = 100%) (Fig. 4).

### TB prevalence according to geographical region

#### TB prevalence in Khartoum State

**Fig. 5:**
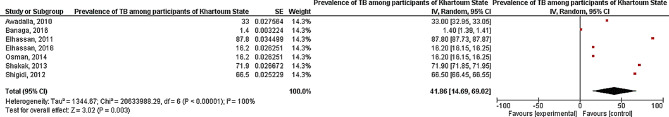
Meta analysis of prevalence of TB among TB patients’ contacts

TB prevalence among residents of Khartoum State was assessed among participants in seven included studies. Studies were conducted among TB patients’ contacts, HIV/AIDS patients, hemodialysis patients and other suspected participants, representing a total sample size of 2,737 participants from both genders in the majority of studies. Participants’ were in their thirties in the majority of studies, while two studies were concerned with children as well as adolescents. The pooled prevalence was 41.86% [CI: 14.69, 69.02]. Heterogeneity was high (I^2^ = 100%) (Fig. 5).

### TB prevalence in Kassala State

**Fig. 6:**
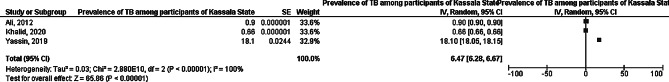
Meta analysis of prevalence of TB among participants from Kassala State

TB prevalence among residents of Kassala State was assessed among participants in three included studies. Studies were conducted among general population as school adolescents and other at risk groups such as pregnant women or suspected patients, representing a total sample size of 5,595 participants as female only in two studies while the third study recruited 2,568 school adolescents from both genders [12]. Participants’ age was described as from 5 to 15 years in a study, mean as 30 years in a study while it was not determined in the third study. The pooled prevalence was 6.47% [CI: 6.28, 6.67]. Heterogeneity was high (I^2^ = 100%) (Fig. 6).

**Table 2:** Summary of prevalence estimates synthesized from tuberculosis related included studies

Prevalence	Assessed in (State)	Assessed among	Total sample size	References	Pooled prevalence	[95% CI]
Prevalence among different populations	Khartoum, Gezira, Kassala, Blue Nile, River Nile, White Nile, Gadarif, Red sea, North Kordofan, Northern State, Sennar & West Darfur	Suspected patients, HIV/AIDS patients, Hemodialysis patients, school adolescents, TB patient contacts, general population & pregnant women	11,153	Agab Eldour, 2014 Ali, 2012 Aman, 2017 Awadalla, 2010 Banaga, 2016 Bottieau, 2022 Elhassan, 2011 Elhassan, 2016 Khalid, 2020 Osman, 2014 Osman, 2017 Shakak, 2013 Shigidi, 2012 Shuaib, 2018 Sirelkhatim, 2016 Yassin, 2019	30.72%	[30.64, 30.81]
Prevalence of pulmonary tuberculosis	Khartoum, Gezira, Kassala, Blue Nile, River Nile, White Nile, Gadarif, Red sea, North Kordofan, Northern State, Sennar & West Darfur	Suspected patients, TB patients’ contacts, HIV/AIDS patients, hemodialysis patients, School adolescents & pregnant women	11,253	Abdallah, 2015 Banaga, 2016 Elamin, 2017 Elmadhooun, 2017 Shigidi, 2012	56.74%	[11.25, 102.23]
Prevalence of extra-pulmonary TB	Kassala, Gadarif, Blue Nile & Khartoum	Hemodialysis patients & suspected patients	2,871	Abdallah, 2015 Banaga, 2016 Elamin, 2017 Shigidi, 2012	13.67%	[1.08, 26.27]
Prevalence of TB among Khartoum State residents	Khartoum	TB patients’ contacts, HIV/AIDS patients, hemodialysis patients & suspected patients	2,737	Awadalla, 2010 Banaga, 2016 Elhassan, 2011 Elhassan, 2016 Osman, 2014 Shakak, 2013 Shigidi, 2012	41.86%	[14.69, 69.02]
Prevalence of TB among Kassala State residents	Kassala	General population, school adolescents, pregnant women & suspected patients	8,163	Ali, 2012 Khalid, 2020 Yassin, 2019	6.47%	[6.28, 6.67]
Prevalence of TB among suspected patients	Khartoum Kassala, Gadarif, North Kordofan & Red Sea	Suspected patients	4,658	Agab Eldour, 2014 Ali, 2012 Bottieau, 2022 Elhassan, 2011 Elhassan, 2016 Osman, 2014 Shuaib, 2018 Sirelkhatim, 2016	33.76%	[25.47, 42.06]
Prevalence of TB among TB patients’ contacts	Eastern Sudan, Khartoum & Gezira	TB patients’ contacts	1,709	Aman, 2017 Osman, 2017 Shakak, 2013	25.40%	[5.67, 45.13]

### Socio-cultural risk factors of TB

#### Gender

Gender was investigated as a possible risk factor toward Tuberculosis in 5 included studies; participants were from Kassala, Khartoum and River Nile States, with 1525 total sample size of male TB and HIV/AIDS patients. The pooled odd ratio of them being infected was 1.81 [1.46, 2.24] with significant p value z = 5.45 (*P* < 0.00001). On the other hand, 822 female were investigated among the same population. The pooled odd ratio of them being infected was 0.55 [0.42, 0.72] with significant p value z = 4.33 (*P* < 0.0001). All results are depicted in (Table 3).

#### Education

Education was investigated as a possible socio-cultural risk factor toward Tuberculosis infection in 5 included studies; participants were suspected TB, *HIV* or *TB* patients from Kassala and River Nile States. Among 1643 participants described as secondary educated or below, the pooled odd ratio of their infection was 0.66 [0.01, 32.48] with insignificant p value z = 0.21 (*P* = 0.84). On the other hand, among 638 participants described as above secondary educated in the same populations, the pooled odd ratio of them being infected was 0.00 [0.00, 2519.91] with insignificant p value as well, z = 0.85 (*P* = 0.40). All results are depicted in (Table 3).

#### Marital status

Marital status was investigated as a possible socio-cultural risk factor toward Tuberculosis infection in 4 included studies; participants were HIV or TB patients from Kassala and River Nile States. Based on a total sample size of 340 single participants, the pooled odd ratio of them being infected was 1.33 [0.34, 5.29] with insignificant p value z = 0.41 (*P* = 0.68). On the other hand, 660 married participant were assessed among the same populations, the pooled odd ratio of them being infected was 0.75 [0.14, 4.09] with insignificant p value as well, z = 0.34 (*P* = 0.74). All results are depicted in (Table 3).

#### Residence

Residence was investigated as a possible socio-cultural risk factor toward Tuberculosis infection in 4 included studies; participants were TB patients from Kassala, River Nile, Khartoum and Gezira States. Among which 812 participants were urban residents, the pooled odd ratio of them being infected was 0.66 [0.34, 1.26] with insignificant *p* value z = 1.27 (*P* = 0.20). On the other hand, 1338 participants were rural residents among the same population, the odd ratio of them being infected was 1.82 [1.01, 3.27] with significant *p* value z = 2.01 (*P* = 0.04). All results are depicted in (Table 3).

#### Knowledge about TB

TB knowledge was investigated as a possible socio-cultural risk factor toward Tuberculosis infection in 2 included studies; participants were TB patients from Kassala and River Nile States, 100 participants were described as having good TB knowledge, the pooled odd ratio of them being infected was 1.15 [0.66, 1.98] with insignificant p value z = 0.49 (*P* = 0.62). On the other hand, 363 participants among the same population was described as having poor TB knowledge, the pooled odd ratio of them being infected was 0.85 [0.49, 1.48]with insignificant p value z = 0.57 (*P* = 0.57). All results are depicted in (Table 3).

#### Socio-cultural risk factors of TB

##### Gender

Gender was investigated as a possible socio-cultural risk factor toward Tuberculosis treatment default in 3 included studies; participants were TB patients from Kassala, Khartoum and Gezira States. Among which 857 were males, the pooled odd ratio of them being defaulted from TB treatment was 1.00 [0.69, 1.46] with insignificant p value z = 0.03 (*P* = 0.98). On the other hand, 466 females were investigated among the same population, the pooled odd ratio of them being defaulted from TB treatment was 1.40 [0.50, 3.94] with insignificant p value as well, z = 0.63 (*P* = 0.53). All results are depicted in (Table 3).

**Table 3:** Summary of socio-cultural risk factors estimates synthesized from Tuberculosis related included studies

Risk	Assessed in (State)	Assessed among	Total sample size	References	Pooled OR [95% CI]	Test for overall effect (z score)
Male gender	Kassala, Khartoum & River Nile	TB & HIV/AIDS patients	1525	Abdallah, 2012 Ahmed, 2021 Awadalla, 2015 El-Muttalut & Elnimeiri, 2017 Saeed, 2021	1.81 [1.46, 2.24]	5.45 (*P* < 0.00001)
Female gender	Kassala, Khartoum & River Nile	TB & HIV/AIDS patients	822	Abdallah, 2012 Ahmed, 2021 Awadalla, 2015 El-Muttalut & Elnimeiri, 2017 Saeed, 2021	0.55 [0.42, 0.72]	4.33 (*P* < 0.0001)
Secondary education and below	Kassala & River Nile	Suspected TB, HIV/AIDS patients & TB patients	1643	Abdallah, 2012 Ali, 2012 Awadalla, 2015 El-Muttalut & Elnimeiri, 2017 Saeed, 2021	0.66 [0.01, 32.48]	0.21 (*P* = 0.84)
Education above secondary	Kassala & River Nile	Suspected TB, HIV/AIDS patients & TB patients	638	Abdallah, 2012 Awadalla, 2015 El-Muttalut & Elnimeiri, 2017 Saeed, 2021	0.00 [0.00, 2519.91]	0.85 (*P* = 0.40)
Single	Kassala and River Nile	TB & HIV/AIDS patients	340	Ahmed, 2021 Awadalla, 2015 El-Muttalut & Elnimeiri, 2017 Saeed, 2021	1.33 [0.34, 5.29]	0.41 (*P* = 0.68)
Married	Kassala and River Nile	TB & HIV/AIDS patients	660	Ahmed, 2021 Awadalla, 2015 El-Muttalut & Elnimeiri, 2017 Saeed, 2021	0.75 [0.14, 4.09]	0.34 (*P* = 0.74)
Urban residence	Kassala, River Nile, Khartoum & Gezira	TB patients	812	Abdallah, 2012 Ahmed, 2021 El-Muttalut & Elnimeiri, 2017 Saeed, 2021	0.66 [0.34, 1.26]	1.27 (*P* = 0.20)
Rural residence	Kassala, River Nile, Khartoum & Gezira	TB patients	1338	Abdallah, 2012 Ahmed, 2021 Ali, 2012 El-Muttalut & Elnimeiri, 2017 Saeed, 2021	1.82 [1.01, 3.27]	2.01 (*P* = 0.04)
Good knowledge about TB	Kassala & River Nile	TB patients	100	Ahmed, 2021 Saeed, 2021	1.15 [0.66, 1.98]	0.49 (*P* = 0.62)
Poor knowledge about TB	Kassala & River Nile	TB patients	363	Ahmed, 2021 Saeed, 2021	0.85 [0.49, 1.48]	0.57 (*P* = 0.57)
Illiteracy	Kassala	TB patients	159	Ahmed, 2021		
Education below secondary	Kassala	TB patients	239	Abdelhadi, 2015		
Secondary education and above	Kassala	TB patients	62	Abdelhadi, 2015		
Not working	River Nile	TB patients	37	Saeed, 2021		
TB treatment Default						
Male gender	Kassala, Khartoum & Gezira	TB patients	857	Abdelhadi, 2015 Ali, 2016 Ismail, 2016	1.00 [0.69, 1.46]	0.03 (*P* = 0.98)
Female gender	Kassala, Khartoum & Gezira	TB patients	466	Abdelhadi, 2015 Ali, 2016 Ismail, 2016	1.40 [0.50, 3.94]	0.63 (*P* = 0.53)

## Discussion

To our knowledge, this review is the first attempt to find out the magnitude of information on pooled prevalence of *TB* as well as its associated socio-cultural risk factors in Sudan. A widespread search from several published databases and stringent methodology to screen and include every potential study was approached in the present study.

Tuberculosis (TB) is a major health problem, with an estimated 10 million people (range 9 to 11.1 million) developing TB disease in 2018, of which 5.8 million, 3.2 million, and 1 million were men, women and children, respectively [34].

The pooled prevalence of TB in the current study was 30.72% and 28.74% after conducting sensitivity analysis. While Extra-Pulmonary *TB* was 13.97%. This finding is much lower than what has been reported in China. China has one of the highest burdens of *TB* in the world, according to the World Health Organization (WHO), the number of new *TB* cases was about 833,000 in China in 2019 [35]. However, a retrospective study conducted in Indonesia where total of 67,944 records were reviewed, the prevalence of *TB* was as low as 0.8% among general population [36]. Moreover, prevalence of *TB* in neighboring Ethiopia- one of the *TB* endemic areas is reported to be 16.7% among random populations [37]. Furthermore, much higher prevalence estimates have been reported in Egypt where it was reported as 70.87% for females and 29.13% for males in a study conducted in Assiut, Egypt [38]. However, the fact that the pooled prevalence synthesized in the current study is based on different study populations of varying infection risk is to be considered when interpreting the current finding.

Moreover, in concern with Extra-Pulmonary *TB* and agreeing to some extent with the current finding; WHO stated that among 6.3 million new *TB* cases recognized in 2017, 16% were extra-pulmonary TB cases; prevalence ranged from 8% in the Western Pacific Region up to 24% in the Eastern Mediterranean Region [39].

Regarding *TB* prevalence among *TB* patients’ contacts; the current study indicated a pooled prevalence of 25.40% [CI: 5.67, 45.13] based on a total sample size of 1,709 participants. However, a related survey was conducted targeting 69,054 populations from 43 villages in Tiruvallure district, India during 2015–2018. This survey indicated a low incidence of 307 per 100,000 [40].

The pooled odd ratio of male being *TB* infected was 1.81 [1.46, 2.24] with significant association in the current study. However, a large study conducted in Morocco reported that the top three contributing risk factors were malnutrition, smoking and HIV infection [41]. Nevertheless, aligning with the current study; several studies indicated that male gender is significantly associated with *TB* infection [42–44].

The strengths of this review are that we systematically identified and included related studies from 2010 to 2022. Moreover; we have conducted meta-analysis to derive pooled prevalence estimates of studies related. Furthermore, we carried out a quality assessment of the included studies based on criteria specifically developed to determine the quality of included studies.

Nevertheless, several limitations are to be considered when interpreting study results; grey literature evidence was not assessed. Moreover, African journals that are not indexed in the screened databases was not considered for inclusion as well, although all included studies are of good quality, several good studies might have been missed. Moreover, publication bias cannot be completely rolled out due to the relatively small amount of published data. Lastly, as a result of the inclusion of publications describing different patient cohorts, the heterogeneity was high among the Meta analysis conducted.

## Conclusion

The current study findings indicate that the pooled prevalence of *TB* is around 30%. Moreover, male gender and rural residence were found to be significantly associated with TB infection. Further research with larger sample sizes targeting prevalence and risk factors of *TB* among Sudanese population is needed to be conducted.

### Electronic supplementary material

Below is the link to the electronic supplementary material.

**Supplementary Material 1: Table (S1):** PRISMA checklist of included studies

## Data Availability

The datasets used and/or analysed during the current study available from the corresponding author on reasonable request.
